# Pharmacogenomics Biomarkers of Soft Tissue Sarcoma Therapies

**DOI:** 10.3389/fonc.2020.00509

**Published:** 2020-04-15

**Authors:** Chiara Caruso, Cecilia Garofalo

**Affiliations:** Advanced Translational Research Laboratory, Veneto Institute of Oncology IOV – IRCCS, Padua, Italy

**Keywords:** soft tissue sarcoma (STS), pharmacogenomics (PGx), resistance and mutation, genetic variation, somatic mutation, toxicity

## Abstract

Soft tissue sarcomas (STS) are heterogeneous rare malignancies comprising ~1% of all solid cancers in adults and including more than 70 histological and molecular subtypes with different pathological and clinical development characteristics. Over the last two decades, the increased knowledge of the new molecular and genomic mechanisms of different STS histotypes allowed for a reclassification of these tumors and consequently to the development of novel chemotherapeutic agents. Generally, surgery, in combination with radiotherapy only in selected cases of localized disease, represents the most common treatment of primary STS, whereas the principal treatment modality for locally advanced or metastatic disease is first-line chemotherapy. The principal treatment for the preponderance of STS patients is usually an anthracycline (epirubicin and doxorubicin) in monotherapy or in combination with other drug novel chemotherapeutic agents. However, survival for treated patients with metastatic disease is poor, and a 2-years survival rate is about 30%. In this scenario, Pharmacogenomics (PGx) biomarkers that can predict drug response play an important role in the improvement of molecular diagnostics in clinical routines and contribute to elucidating the genetic basis for the differences in treatment efficacy and toxicity among STS patients. This review focuses on recent insight in the PGx biomarkers that have been described to modulate responsiveness and toxicity parameters of conventional and new chemotherapeutics drugs in several STS histotypes.

## Introduction

Soft tissue sarcomas (STS) are heterogeneous rare malignancies representing of about 1% of all solid tumors in adult and including more than 70 histological and molecular subtypes with a multiplicity of pathogenic and clinical development features (1–4). STSs origin from mesenchymal cells of a variety of tissue lineage, including adipose, muscle, fibrous cartilage, and vasculature. Among this heterogeneousness, the most common STS is represented by liposarcoma (LPS), accounting for one-fourth of all extremity STS and half of retroperitoneal STS (5). Different histotypes of high-grade STS frequently diagnosed include leiomyosarcoma, synovial sarcoma, undifferentiated pleomorphic sarcoma, and malignant peripheral nerve sheath tumors.

Over the last two decades, the increased knowledge of the new primary molecular and genomic mechanisms of different STS histotypes allowed to a reclassification of these tumors and consequently to the discovery of innovative chemotherapeutic agents (6). Overall, sarcomas can be classified in two comprehensive genetic groups depending on the chromosomal aberration occurring in the genome: those harboring specific genetic alterations like activating mutations and/or translocations showing simple karyotypes (which represent almost 30% of all sarcomas) and those with more complexity on karyotypes (7). The gene fusions resulting from specific translocations encoding chimeric transcription factors affecting transcriptional regulation of target genes are frequently detected in sarcomas, while others encode chimeric growth factors or protein tyrosine kinases (8).

Despite the prominent progress in discovering genetic aberrations and their functions in STS, the major therapeutic modality for most local recurrence and metastatic sarcomas remains cytotoxic chemotherapy. Generally, surgery, in combination with radiotherapy only in selected cases of localized disease, represents the most common treatment of primary STS, whereas the principal treatment modality for locally advanced, or metastatic disease is chemotherapy. First-line drug protocol for the preponderance of STS patients is usually an anthracycline (epirubicin and doxorubicin) alone or in combination with another drug (9, 10). However, survival for treated patients with metastatic disease is only 14-17 months, and 2-years survival rate is about 30%.

Therefore, there is an urgent need to develop novel treatments and find biomarkers that can help physicians to identify patients who are possible good responders or resistant to specific therapies and predict individual predisposition to toxicity reactions associated with therapies.

Differences in pharmacological response to drugs represent the most common cause of patient morbidity and mortality. From this specific point of view, pharmacogenomics (PGx) biomarkers that can predict drug response play an important role in the improvement of molecular diagnostics in clinical routines and contribute to elucidating the genetic basis for the differences in treatment efficacy and toxicity among patients. Moreover, PGx markers predicting efficacy or risk to develop adverse drug reactions (ADRs) are commonly positioned in transporters, drug-metabolizing enzyme genes, drug targets, or HLA alleles.

Genetic variability harboring in the germline genome of the patient can influence systemic pharmacokinetics and pharmacodynamics of the treatments, acting as prognostic biomarkers for drug-induced toxicity and treatment efficacy. Instead, the aberrations in cancer somatic genome mostly function as drug targets and they can be used to select treatment or to be predictive of response to treatment (11).

Very penetrant predisposed mutations and frequent genetic variants particularly single-nucleotide polymorphisms (SNPs) which heredity pass between the generations represent the mostly germline variations that are considered as useful biomarkers for ADR and drug response. Contrary, due to exposure to chemotherapeutics that likely act through damage to DNA, cells could acquire randomly somatic mutations that are potentially used as drug targets (12, 13).

Thanks to significant improvements in biotechnology and bioinformatics knowledge, genomic research quickly advanced from investigations based on modifications at the single gene level to studies on the whole-genome scale using extensive genotyping, and Next Generation Sequencing (NGS) methods. These new methodologies considerably decrease sequencing times and costs and allow early detection of disorders and identification of pharmacogenomics markers to customize treatments (12, 14).

Candidate gene methods are performed to recognize most of the germline variations while genome-wide association (GWAS) approach is archived sequencing up to a large number of SNPs. On the other hand, somatic mutations from cancer genomes are analyzed through NGS technique that uses the concurrent sequencing of a huge number of DNA parts to create an enormous pool of genomic arrangement information. This procedure allowed genotyping a selected number of the gene of interest (gene panel), the complete exome or the whole genome.

In this review, we outline recent studies on PGx biomarkers that have been described to modulate responsiveness and toxicity parameters of conventional and new option chemotherapeutics drugs in several STS histotypes (Tables 1, 2).

**Table 1:** Germline variants biomarkers in STS therapies.

**Drug**	**Gene**	**Germline biomarker**	**STS histotype**	**References**
Trabectedin	BRCA1	AAAG rs16941	Advanced STS	(18)
	BRCA2	(LOH) rs80359030	Uterine Stromal Sarcoma	(20)
Imatinib	VEGFR2	AA rs1870377	GIST	(27)
	VEGFA	AA rs1570360		
	SLCO1B3	T rs4149117		
Sunitinib	POR	TT rs1056878	GIST	(28)
	SLCO1B3	T rs4149117		
	SLC22A5	C rs2631367		

**Table 2:** Pharmacogenomics (PGx) somatic biomarkers in STS therapies.

**Drug**	**Gene**	**Somatic variation**	**STS histotype**	**References**
Pazopanib	TP53	mutation	Advanced STS	(33)
Gemcitabine	hENT1	high expression	Leiomyosarcoma; Angiosarcoma	(38)
Trabectedin	BRCA1	low expression	Leiomyosarcoma; Myxoid Liposarcoma; Liposarcoma; Osteosarcoma; Synovial Sarcoma; Uterine Leiomyosarcoma; Ewing Sarcoma	(39)
	ERCC5/XPG	high expression	Leiomyosarcoma; Myxoid Liposarcoma; Liposarcoma; Osteosarcoma; Synovial Sarcoma; Uterine Leiomyosarcoma; Ewing Sarcoma	
Conventional chemotherapies	CD109 (TGF-β)	high expression	Myxofibrosarcoma	(44)
Conventional chemotherapies	RB1; CDKN2A; CDKN2B; CCND1; CDK6; TP53	mutation	Myxofibrosarcoma	(45)
	KRAS	amplification		

## Germline Variants as Potential Biomarkers for Drug Response

Several germline biomarkers could impact on effectiveness of therapies and survival in STS patients and may be useful to stratify patients liable to develop treatment-associated toxicities.

One of the new therapeutic alternatives among the few options of STS treatments is trabectedin (Yondelis) a marine-derived compound extracted from the Caribbean Sea squirt *Ecteinascidia turbinate*.

In phase III clinical trial in advanced leiomyosarcoma and liposarcoma patients showing progression disease after anthracycline-based chemotherapy, trabectedin significantly increases disease control respect to conventional dacarbazine treatment (15).

Several studies confirmed that the cytotoxic activity of trabectedin toward cells has been associated with the peculiar capacity to modify positively the tumor microenvironment and exert strong immunomodulatory effects (5, 16). The main antiproliferative mechanism consists of transcription regulation and DNA repair systems, including transcription-coupled nucleotide excision repair (TC-NER), homologous recombination repair (HRR) and, DNA repair genes such BRCA1 (BReast-CAncer susceptibility gene 1) and BRCA2. Additionally, the association of BRCA mutational status with improved clinical response to trabectedin explains the specific sensitivity of STS patients to this drug. Several clinical studies confirmed an improved prognosis and overall survival in patients carrying germline mutation or absence of BRCA compared to non-carriers (17). *Italiano et colleagues* have pointed out the relationship of precise haplotypes associated with trabectedin sensitivity to specific SNPs within the BRCA1 gene (18). In this study, advanced STS harboring at least one AAAG allele on BRCA1's haplotype displayed a statistically significantly longer progression-free survival (PFS) and overall survival, compared with STS without AAAG allele. Moreover, in 29% of human uterine leiomyosarcoma one of the histotypes more responsive to trabectedin, BRCA1 protein was not express (19).

A remarkable clinical case study describes a patient with advanced uterine stromal sarcoma with bone and hepatic metastases carrying a specific BRCA2 germline variant. The authors revealed a complete rapid response following trabectedin treatment linking this positive effect to the loss of heterozygosity (LOH) of the mutated BRCA2 gene. These analyses corroborate the assumption that different DNA repair defects existing in tumors positively conditioned the response to trabectedin and that BRCAness malignant genotype is significant in influencing the effectiveness of treatment including trabectedin (20).

Gastrointestinal stromal tumors (GIST) are the most prevalent tumors of the gastrointestinal tract origin from mesenchymal lineage (21). Mutation in tyrosine protein kinase KIT and platelet-derived growth factor receptors (PDGFRA) genes are present in 75–80% and 5–10% of GISTs, respectively with their consequent constitutive activation. Imatinib, sunitinib and regorafenib, TKIs that inhibit KIT/PDGFRA tyrosine kinase, demonstrated efficacy in unresectable and/or metastatic GIST (22). In almost 80% of patients with advanced or metastatic GIST treated with imatinib (400 mg per day), quick partial response or stable disease was observed for ~18–36 months, with some patients in therapy for 10 years. Despite the greater clinical advantage of these drugs, PFS is variable due to a frequent resistance mechanism depending on mutational board of KIT/PDGFRA genes. Commonly, GISTs harbor KIT mutation in exon 11 and less frequently in exon 13 in imatinib-naïve patients, while exon 9 mutation reduces sensitivity and the rare KIT exon 17 mutations (e.g., D816V) exert resistance to imatinib. Moreover, the common D842V mutation in PDGRFA gene is correlated to imatinib, sunitinib, and regorafenib resistance, whereas wild-type GISTs negative for KIT/PDGRFA mutations are insensitive to imatinib (23–25). Thus, it is crucial to find novel prognostic biomarkers to stratify patients with improved risk for disease progression during imatinib therapy. Analysis of SNPs variant in *VEGFRA2, VEGFA*, and Solute Carrier Organic Anion Transporter Family Member 1B3 (*SLCO1B3)* display a correlation of these SNPs with PFS in patients with advanced GIST receiving imatinib (26). Genetic variant analysis of 36 SNPs in 18 genes performed in patients with advanced GIST treated with imatinib demonstrated a correlation between worse PFS and VEGFR2, VEGFA, and *SLCO1B3* carrying specific genotype listed in Table 1 (27).

Association of SNP and outcome of GIST patients cured with sunitinib was also highlighted by *Kloth and colleagues*. In this study, PFS and OS in 127 patients with advanced GIST treated with sunitinib were associated with 49 SNPs involved in the pharmacokinetic and pharmacodynamic pathway of this TKI. More specifically, PFS was significantly extended in carriers rs1056878 (TT genotype) in *Cytochrome p450 oxidoreductase* (*POR*). Otherwise, the presence in patients carrying the T-allele in SLCO1B3 rs4149117, the CCC-CCC alleles in SLC22A5 haplotype, and the GC-GC alleles in the IL4 R haplotype were predictive for OS (28).

Pazopanib, currently approved for the treatment of different STS, is multitarget TKI exerting its clinical antitumor effects through inhibiting vascular endothelial growth factor receptor (VEGFR)-mediated angiogenesis and by directly blocking PDGFRs, fibroblast growth factor receptors (FGFRs), and KIT (29, 30). The results of the PALETTE study designed to compare the efficacy and safety of pazopanib with placebo in advanced pretreated STS, led to Pazopanib, approval as single-agent in patients with metastatic STS from non-adipocytic origin (31). One of the potentially serious consequences of TKI therapy usually described in patients following pazopanib therapy is hepatotoxicity. Recent data provide innovative understanding connecting the pazopanib-associated hepatotoxicity to an immune-mediated mechanism in some patients, demonstrating that HLA-B^*^57:01 allele carriage is correlated with elevated ALT values in these patients and identifying genetic PGx predicting liver damage (32).

## Somatic Mutation Biomarkers

Genetic analysis of STS shows low mutational load including predominantly by copy number changes (6). Whole-exome sequencing (WES) data analysis of 206 sarcomas of different histotypes identifies TP53, ATRX, and RB1 significantly mutated genes across sarcoma histotypes where TP53 mutations were most prevalent in leiomyosarcoma and RB1 mutations were seen in leiomyosarcoma, undifferentiated pleomorphic sarcoma, and myxofibrosarcoma (6).

A recent retrospective study reported the new early PGx markers related to response and toxicity of pazopanib therapy in advanced STS. In this study, application of NGS analysis performed to sequence several genes related to cancers in pretreatment tumor specimens from patients with advanced STS treated with antiangiogenic agents (pazopanib and sunitinib) (33), reveals the importance of TP53 and RB1 genes in modulating the outcome of TKI treatments. Although all loss-of-function mutational status of TP53 detected (missense mutation of DNA binding and/or tetramerization domain, or homozygous deletion) was not correlated to outcome of patients treated with pazopanib, *TP53* mutations were shown to have significant association with a longer PFS respect to *TP53* wild-type. Predictors factors of pazopanib effectiveness and toxicity in STS patients are associated also with modulation of cytokines and circulating angiogenic factors in serum (34). Indeed, PFS observed after 12 weeks of treatment was positively correlated to high levels of interleukin (IL)-12 and mitochondrial pyruvate carrier 3 (MPC3) levels at baseline, and negatively associate with low soluble VEGFR2 and high placental growth factor (PGF) levels.

Gemcitabine, in monotherapy or combined with docetaxel, has been usually approved in leiomyosarcoma (35) and angiosarcoma (36) treatments.

Intracellular uptake of prodrug gemcitabine into tumoral cells takes place through a transmembrane protein human equilibrative nucleoside transporter 1 (hENT1) (37). A recent retrospective analysis demonstrated that positive clinical outcome of leiomyosarcoma (PFS: 6.8 vs. 3.2 months; OS: 14.9 vs. 8.5 months) and angiosarcoma (PFS was 9.3 vs. 4.5 months; OS 20.6 vs. 10.8 months) patients treated with gemcitabine was linked to high hENT1 tumor expression levels (38). Thus, since the identification of molecular markers like hENT1 could predict gemcitabine efficacy in leiomyosarcoma and angiosarcoma patients, evaluation of hENT1 expression level would allow a better patient selection with a high possibility to benefit from this chemotherapy regimen.

Not only germline variants as discussed before but also somatic alterations in the homologous repair system are reported to be responsible for a deeper and longer activity of trabectedin in STS patients where drug response is inversely correlated with the BRCA1 mRNA levels (39). In this clinical report, the investigators established that low levels of mRNA BRCA1 expression statistically significant associate with an improved outcome of patients in terms of disease control rate (48 vs. 26%, *p* < 0.01) and longer median survival (15.4 vs. 7.1 months, *p* < 0.002). Interestingly, patients with decreased level of mRNA BRCA1 showed a better median PFS (4.7 vs. 2.0 months, *p* = 0.002) and a progression-free at 6-months (PFS-6) after treatment (43 vs. 23%, *p* < 0.012). Moreover, a significant correlation between increased responses to trabectedin treatment with high expression level of ERCC5/XPG complex was also observed in patients showing an improvement in term of disease control rate (56 vs. 36%, *p* = 0.04), median PFS (7.1 vs. 2.5 months, *p* = 0.002), and PFS after 6 months after trabectedin therapy (52 vs. 30%, *p* = 0.01). These data support the hypothesis of a direct association between DNA damage repair system functionality and responsiveness to trabectedin, differently from other DNA interacting agents.

In myxofibrosarcoma, a common adult STS characterized by a high local recurrence rate and infiltrative growth pattern surgery combined with neoadjuvant or adjuvant radiotherapy represent the standard care in localized disease (40–42). However, chemotherapy treatment is considered for metastatic myxofibrosarcoma despite the outcome remains very poor and identification of PGx markers is still limited (43).

Genotyping analysis in patient-derived MFS primary cultures demonstrated the promising role of surface glycoprotein CD109, a negative regulator of transforming growth factor-beta (TGF-β) pathway in the differential diagnosis of more aggressive high-grade myxofibrosarcoma identifying this marker as a possible therapeutic target (44). Moreover, in this study, the authors highlighted the value of TGF-β expression as an advantageous marker for chemotherapy efficacy and resistance. Indeed, in patient-derived colture cells of myxofibrosarcoma, the expression of TGF-β was negatively correlated to sensitivity to treatments.

In an extensive integrated genetic and epigenetic study of 99 myxofibrosarcoma performed by WES, RNA sequencing, and methylation analysis, a large number of driver genes were identified as potential drug targets and molecular prognostic factors in this STS histotype (45). This study demonstrated the association of the mutational board of cell cycle regulators (*RB1, CDKN2A, CDKN2B, CCND1*, and *CDK6*) with a worse overall survival as well as *TP53* alteration and *KRAS* amplification. Thus, considering as PGx markers in a specific subset of these tumors genetic alterations in the Rb pathway, comprising *CCND1* or *CDK6* amplification, these data will contribute to knowledge for the use of novel therapeutic approaches such as CDK4/6 inhibitors.

Besides genetic factors, epigenetic modifications of DNA together with miRNA regulation of gene expression have been linked to differences in drug response, through regulation key drug-metabolizing genes or increasing expression of drug efflux transporters (46–49).

The role of these biomarkers in mediating chemotherapy efficacy was underlined in eribulin-based therapies in STS patients. Eribulin mesylate is a microtubule inhibitor equivalent to halichondrin B derivate from the marine sponge *Halichondria okadai*. The inhibition of tubulin by eribulin induces G2/M cell-cycle arrest, disruption of mitotic spindles, and, finally, apoptosis. Patient-derived primary coltures of adipocytic and undifferentiated pleomorphic sarcoma demonstrated high sensitivity to eribulin (50, 51). Moreover, the antitumor activity of eribulin in metastatic STS patients was confirmed in recent EORTC 62052 phase II and III clinical trials (52–54). miRNA expression signature in 65 tumor samples from patients included in the EORTC phase II clinical trials indicated miR 106a, miR-17, and miR-34a as markers modulated in eribulin responders respect to non-responders STS patients, pointing out the role of these miRNA as useful tools for clinical practice to stratify patients that can really benefit from the eribulin treatment (55).

## Discussion

Pharmacogenomics studies of anti-cancer drugs in STS play an important role in identifying patients avoiding adverse events, and optimizing drug dose. The aim of these investigations is to take advantage of personalized chemotherapies regarding cancer treatment and prevention. Development in NGS technologies has been open a new opportunity for characterizing the genomic landscape of these tumors, together with the possibility of applying the genetic diagnostic tests relevant in cost-benefit analysis. However, due to the several rare STS histological subtypes harboring specific fusion genes (56), certain limitation should be considered for the most of the studies on NGS analyses that consider together samples from different STS histotypes where panel with a limited number of covered genes are used. In this particular point of view, the implementation of a panel containing an increased number of genes seems to be mandatory for a better daily diagnostic routine in STS.

Finally, future studies in this field should be considered in terms of identification and validation of drug-sensitivity test systems for routine use that include known specific PGx markers in common clinical management.

## Author Contributions

CC contributed to references collection of the study. CG contributed to the writing of this manuscript. All authors approved the final version.

### Conflict of Interest

The authors declare that the research was conducted in the absence of any commercial or financial relationships that could be construed as a potential conflict of interest.
